# Effects of support and reaction pressure for the synthesis of dimethyl ether over heteropolyacid catalysts

**DOI:** 10.1038/s41598-020-65296-3

**Published:** 2020-05-22

**Authors:** Cristina Peinado, Dalia Liuzzi, Rosa María Ladera-Gallardo, María Retuerto, Manuel Ojeda, Miguel A. Peña, Sergio Rojas

**Affiliations:** Grupo de Energía y Química Sostenibles (EQS). Instituto de Catálisis y Petroleoquímica CSIC. C/Marie Curie 2, 28049 Madrid, Spain

**Keywords:** Heterogeneous catalysis, Biofuels

## Abstract

Dimethyl ether (DME) is an advanced second-generation biofuel produced via methanol dehydration over acid catalysts such as γ-Al_2_O_3_, at temperatures above 240 °C and pressures above 10 bar. Heteropolyacids such as tungstosilicic acid (HSiW) are Brønsted acid catalysts with higher DME production rates than γ-Al_2_O_3_, especially at low temperatures (140–180 °C). In this work, we show that the performance of supported HSiW for the production of DME is strongly affected by the nature of the support. TiO_2_ and SiO_2_ supported HSiW display the highest DME production rates of *ca.* 50 mmol_DME_/h/g_HSiW_. Characterization of acid sites via ^1^H-NMR, NH_3_-isotherms and NH_3_-adsrobed DRIFT reveal that HSiW/X have Brønsted acid sites, HSiW/TiO_2_ showing more and stronger sites, being the most active catalyst. Methanol production increases with T until 200 °C where a rapid decay in methanol conversion is observed. This effect is not irreversible, and methanol conversion increases to *ca.* 90% by increasing reaction pressure to 10 bar, with DME being the only product detected at all reaction conditions studied in this work. The loss of catalytic activity with the increasing temperature and its increasing with reaction pressure accounts to the degree of contribution of the pseudo-liquid catalysis under the reaction conditions studied.

## Introduction

The production of dimethyl ether (DME) from biomass is attracting a great deal of attention in industry and academia. DME is mainly produced from natural gas or coal-derived methanol via dehydration processes^1^. DME can be also produced from biomass-derived syngas thus being considered a second-generation biofuel that can be used as diesel substitute or blend or as substitute for liquefied petroleum gas (LPG)^2,3^. DME is a gas at ambient temperature and pressure, but liquefies at 5.9 bars and 25 °C^4^, which is advantageous in terms of storage and handling. It is a non-toxic, non-dangerous chemical. Moreover, it is rapidly photodegraded to H_2_O and CO_2_ if released to the atmosphere. DME is widely used as aerosol propellant and due to its physical properties such as flammability and stability are similar to that of LPG, so it is used as LPG blend or as fuel for power generation in gas turbines^1^. In recent times, however, the interest in DME has shifted and due to properties such as high cetane number between 55–60, low auto-ignition temperature and its low emissions of soot DME is currently viewed as an ideal diesel substitute or blend.

DME is typically obtained via methanol dehydration (2CH_3_OH → CH_3_OCH_3_ + H_2_O) in gas phase over acid catalysts such as γ-Al_2_O_3_, zeolites or silica-modified alumina at moderate pressures between 10 and 20 bar, and temperatures 250–300 °C^1,5–14^. However, under such conditions undesired products such as olefins are produced and react further to coke species, thus leading to a rapid deactivation of the catalyst. It has been recently demonstrated that TiO_2_ supported heteropolyacids (HPA) exhibit higher reaction rates in methanol dehydration reaction than γ-Al_2_O_3_ under milder reaction conditions (1 bar and 180 °C) with 100% selectivity for DME^15–17^, thus preventing catalyst deactivation by coke formation/deposition.

The Keggin structure is one of the most common and best known types of HPA structures for catalytic applications, as well as the best-known one. It is formed by four units of three octahedrons (MO_6_) stabilized by protons, that surround a central tetrahedral unit (XO_4_). They are usually formulated as H_8-n_[X^n+^M_12_ O_40_], where X is the central atom, n is the oxidation state and M is the metal ion. This arrangement constitutes the primary structure of the HPA, while the way in which these units arrange three-dimensionally combined with crystallization water is known as the secondary structure, which may be flexible. The presence of double bonds M = O in these structures results in the delocalization of the negative charge, conferring a high mobility to the protons of the HPAs’ structures. As a consequence, HPAs present strong Brønsted acidity and as a matter of fact the strength and density of the acid sites depend on the content of water, which forms hydrogen bonds. Due to this singular structure, the catalytic behavior of these materials is complex, with active sites located both on the surface and within the solid. Therefore, in addition to the typical heterogeneous catalysis that takes place at surface sites, including pore walls, reactants are absorbed within the bulk, that is, into the interanion space, where they are able to react as well. This latter feature is referred to as bulk-type or pseudo-liquid catalysis^18–20^.

In particular, phosphotungstic acid (H_3_PW_12_O_40_, HPW) and silicotungstic acid (H_4_SiW_12_O_40_, HSiW) have been reported among the most active HPA structures for alcohol dehydration^15–17,21,22^, which have specifically demonstrated to achieve high conversion and selectivity in the DME synthesis reaction^15,16,23^ at temperatures as low as 140–160 °C and atmospheric pressure. For instance, when supported on inorganic carriers such as TiO_2_, methanol conversions as high as 80% and 100% DME selectivity at 200 °C have been reported^15,16,23^. In these works, the beneficial effect of supporting the HPAs on TiO_2_ was clearly demonstrated and TiO_2_ supported HSiW roughly doubles the methanol conversion and DME productivity achieved by non-supported HSiW at temperatures ranging from 140 to 160 °C^16,23,24^. Recent studies have also confirmed the beneficial effect of supporting HPA on carriers such as SiO_2_, BN, and carbon nanotubes^16,24–26^. However, comprehensive investigations about the effect of the support are still lacking in the literature.

The superior performance of supported HSiW and HPW for the synthesis of DME from methanol has been only demonstrated at low temperatures (below 200 °C) and pressures (1 bar). These reaction conditions may not be feasible and economically favorable for the production of DME in large-scale commercial plants, in which methanol production from syngas takes place at high temperatures (240 °C and higher) and pressures (20–70 bar). Moreover, this feature is critical for the direct synthesis of DME from syngas since the reactor must operate at sufficient temperatures and pressures (well above 180 °C and 1 bar) to efficiently transform syngas into methanol (using Cu/ZnO based catalysts) previous to producing DME^27,28^.

In this work, we show that when supported onto inorganic carriers, silicotungstic acid (HSiW) is a more active catalyst for the dehydration of methanol to DME than γ-Al_2_O_3_, producing only DME at all reaction conditions studied in this work. The catalytic performance of the supported HSiW materials for DME production depends on the nature of the support, being the catalyst supported on TiO_2_, ZrO_2_ and SiO_2_ the ones displaying the highest methanol conversions. Whereas methanol conversion remains stable at temperatures below 190 °C, increasing reaction temperature at 200 °C or above leads to a rapid decline of methanol conversion with time on stream without affecting product selectivity. However, methanol conversion increases to its initial value by increasing reaction pressure. This behavior is ascribed to the contribution of pseudo-liquid catalysis under the reaction conditions studied in the manuscript.

## Experimental

### Synthesis of supported HSiW catalysts

Supported HSiW catalysts (HSiW/X, where X is the support) were synthesized following the impregnation at incipient wetness method, consisting in the dropwise addition of a solution of HSiW (H_4_SiW_12_O_40_·xH_2_O, Sigma-Aldrich, ≥99.9%) in ethanol (Panreac) to the corresponding support. Different supports have been studied in this work namely TiO_2_ (Degussa P-25, 80% anatase, 20% rutile), SiO_2_ (BASF D11-11), CeO_2_ (Reacton, Alfa Aesar, 99.99%, 14 Micron Powder), BN (Aldrich, 98%, powder), ZrO_2_ (Sigma-Aldrich, 99%) and Al_2_O_3_ (Condea, Puralox NWa-15). The quantities of HSiW and support were chosen by taking into account that the loading of HSiW in the catalyst should satisfy the relationship 4.5 Keggin units per square nanometer (KU/nm^2^) of the support. This loading has been previously demonstrated to lead to the highest conversion of methanol per gram of catalyst for heteropolyacids supported on TiO_2_^23^. Maintaining constant this value means that the weight percentage of HSiW for each catalyst is different, depending on the specific surface area of the support. After impregnation, the catalysts were held for 24 hours at room temperature and then dried at 60 °C overnight. Catalysts were named as HSiW/X, where X identifies the support. Bulk HSiW and γ-Al_2_O_3_ (Alfa Aesar bimodal 70–5000 Å) were dried and used as reference catalysts.

### Catalyst characterization

The specific surface areas of the catalysts were obtained from the N_2_ adsorption/desorption isotherms using the BET method in an ASAP2020 Micromeritics apparatus. The samples were first degassed at 140 °C and then the N_2_ adsorption/desorption isotherms were collected at −196 °C. The crystalline structure of the catalysts and the supports was determined by X-ray diffraction (XRD), using a powder X-ray X´Pert Pro PANalytical with a configuration θ − 2θ, using CuKα radiation, with an Anton Paar XRK900 for the x-ray diffraction data collected in a range of temperatures between 20 and 550 °C. The crystallite sizes of the HSiW phases on each catalyst were calculated by applying the Scherrer Eq. 1 to the diffractograms collected at 150 °C, since this is the highest temperature at which the structure of the hexahydrated phases (H_4_SiW_12_O_40_·6H_2_O) is stable in all catalysts.

1 $$d=\frac{k\lambda }{{\beta }_{size}\,\cos \,\theta }$$

Where *k* is the Scherrer constant (*k* ≈ 0.94), λ is the incident wavelength, θ is the diffraction angle and β_size_ is the experimental full-width at half-maximum (FWHM). This equation is employed to obtain an estimated value of the size of the X-ray coherent diffracted crystalline domains (*d*).

We determined the mass specific surface areas (*As*) using the size of the particles determined by XRD and approximating to a spherical geometry using Eq. 2^29,30^:

2 $${A}_{S}=Area/Volumen\times Density=6/d\times \rho $$

Where *d* is the diameter of the particles and ρ is the heteropolyacid bulk density calculated for H_16_O_46_SiW_12_·6H_2_O (Pn-3mc, Z = 2, V = 1789.63 Å^3^, Mw = 2986.26 g/mol).

A Bruker AV400-WB spectrometer was used to collect the solid-state NMR spectra of the catalysts, at ^1^H NMR frequency of 400.13 MHz, using a 2.5 mm double-resonance MAS probe. Spinning speed was set at 25 kHz. Single-pulse experiments used a 3 ls p/2 pulse, spectral width of 35 kHz, and a relaxation delay of 2 s. Raman spectroscopy was used to determine the molecular structure of the prepared materials, using a Renishaw in via Raman Microscope spectrometer. It is equipped with a laser beam emitting at 532 nm with a 1800 lines/mm grating monochromator, and other beam emitting at 785/532 nm and 300 mW with a 1200/1800 lines/mm grating monochromator. The scattered photons were simultaneously collected on a CCD camera. The spectral resolution was 1 cm^−1^ using a 50x objective. The number of acid sites of the samples was determined by two NH_3_ adsorption isotherms performed 100 °C with ASAP 2010/2000 Micromeritics equipment. Prior to the second adsorption, the samples were treated at 200 °C under vacuum for 12 hours. The difference between the isotherms corresponds to the irreversibly chemisorbed NH_3_, which was not released during the vacuum treatment. This technique was used to avoid the high temperatures of the temperature programmed desorption of NH_3_ technique, which would destroy the structure of the HPA before the NH_3_ was released. A FTIR-6300 JASCO Fourier transform spectrophotometer equipped with a Harrick diffuse reflectance accessory (HVC-DRP cell) was used to record the DRIFT spectra. Before collecting the spectra, the catalysts were pretreated at 220 °C in a current of He (20 mL min^−1^) during 30 min. NH_3_ was used as probe molecule to identify acid sites on the catalysts. A flow of 20 mL min^−1^ of NH_3_/He (5/95 vol) was passed through the DRIFT cell during for 15 minutes. The cell was purged under a 20 mL min^−1^ flow of He; the spectra were recorded at 100 °C. For methanol adsorption experiments, the methanol was introduced into the DRIFT cell by bubbling He (20 mL min^−1^) through a glass saturator filled with methanol at room temperature during 30 min. After methanol adsorption/reaction, the samples were treated in He flow at increasing temperatures from 140 to 400 °C at 10 °C min^−1^.

### Catalytic tests

The catalytic activity of the bulk and supported HSiW samples in the methanol dehydration reaction was measured in a fixed bed reactor placed into a furnace to control its temperature, which was measured by a thermocouple in contact with the catalytic bed. The catalytic bed is formed by 0.2 g of catalyst diluted in SiC up to 1.8 mL to minimize the formation of significant exotherms in the bed, with a particle size between 250 and 300 µm. Methanol and N_2_ (13% vol. methanol) were fed to the reactor with a HPLC pump and a TOHO TTM 005 mass flow controller, respectively. The methanol was evaporated and mixed with the N_2_ before being introduced into the reactor. The effluents of the reactor were measured *on line* with a Varian CP-3800 gas chromatograph equipped with a SPB-5, 60 m x 0.53 mm capillary column connected to a flame ionization detector (FID) where methanol and DME were separated and analyzed. Catalytic tests were carried out at different pressures (1, 10, 20 and 24 bar), different temperatures (140–240 °C) and maintaining the methanol liquid hourly spatial velocity (LHSV) constant at 1.11 h^−1^. Catalysts were treated *in situ* in a N_2_ flow at 220 °C during one hour prior to each experiment. Methanol conversion and DME production rate were calculated from Eqs. 3 and 4 (note that DME is the only product detected in all reactions studied in this work).

3 $$Methanol\,conversion({X}_{C{H}_{3}OH},\, \% )=\frac{Moles\,of\,methanol\,consumed}{Moles\,of\,methanol\,fed}\cdot 100$$

4 $$DME\,production\,rate\,(mmo{l}_{DME}{h}^{-1}{{g}_{HSiW}}^{-1})=\frac{C{H}_{3}OH\,flo{w}_{in}(mmol\,{h}^{-1})\cdot {X}_{C{H}_{3}OH}( \% )/2}{mas{s}_{HSiW/X}(g)\cdot HSiW\,in\,the\,catalyst\,(wt.\, \% )}$$

Where CH_3_OH flow_in_ represent the number of moles of methanol fed to the reactor per unit of time.

## Results and discussion

### Catalyst characterization

The textural properties of the catalysts and of the supports, along with the actual HSiW loading in each catalyst are shown in Table 1. The isotherms for the HSiW/X and for the bare supports are shown in Supplementary Fig. S1 online. HSiW records a very small specific surface area of 8 m^2 ^g^−1^ with no porosity.

**Table 1:** Composition, textural properties, crystallite sizes and HSiW surface area of HSiW/X catalysts.

Catalyst	HPA content (wt. %)	Surface area (m^2^ g^−1^)		Pore volume (cm^3^ g^−1^)		Pore diameter (nm)		d (nm)	As (m^2^g^−1^)
		Support	Catalyst	Support	Catalyst	Support	Catalyst	Catalyst	
HSiW (bulk)	100	—	8	—	—	—	—		
HSiW/TiO_2_	52	53	40	0.25	0.17	50	52	24	45.1
HSiW/SiO_2_	73	129	42	0.66	0.11	41	45	29	37.3
HSiW/ZrO_2_	46	39	27	0.27	0.04	46	14	17	63.7
HSiW/BN	28	25	15	0.10	0.06	10–100	23, 65	15	72.2
HSiW/Al_2_O_3_	74	147	49	0.44	0.05	9, 12	9	10	108.3
HSiW/CeO_2_	61	74	57	0.21	0.05	47	10	12	90.2

The specific surface area and the pore volume of the HSiW supported catalysts are lower than those of the bare supports, but higher than that of bulk HSiW. The pore diameters of the HSiW/X catalysts are characteristic of each material, but all lie within the mesoporous range, between 2 and 50 nm.

**Figure 1:**
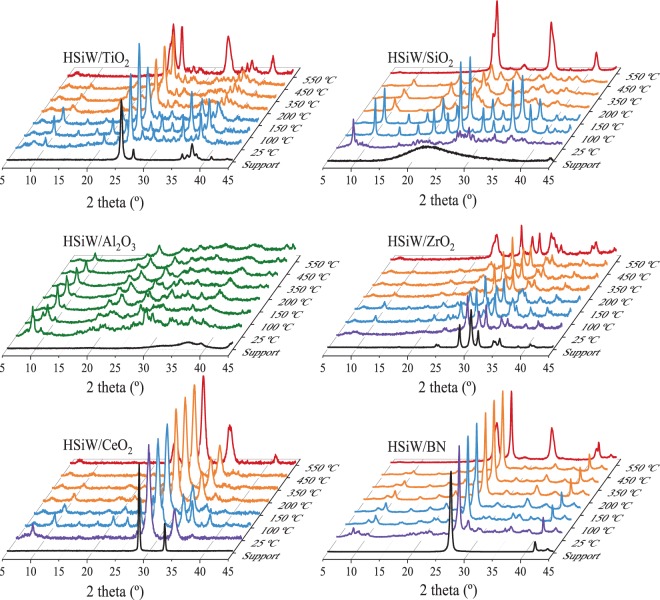
X-ray diffractograms of HSiW/X recorded under air atmosphere between 25 and 550 °C. Purple: H_4_SiW_12_O_40_·(6-21)H_2_O; blue: H_4_SiW_12_O_40_·6H_2_O; orange: H_4_SiW_12_O_40_; red: WO_3_; green: unidentified pattern. The diffractograms for the support are shown as black lines on each set of diffractograms.

In order to investigate if the structure of HSiW has been modified during the impregnation process and to assess the stability of the HSiW phases with temperature, we rely on XRD and Raman analyses. Fig. 1 shows the XRD patterns of the catalysts recorded at different temperatures under a flow of O_2_/N_2_ 20/80 vol. As observed, concerning the HPA phase, the diffractograms of all HSiW/X samples recorded at room temperature show weak and broad reflections corresponding to H_4_SiW_12_O_40_·*x*H_2_O with a water content between 6 and 21 molecules^31,32^, probably H_4_SiW_12_O_40_·14H_2_O, with the most intense reflections around 8 and 29° ^32^. However, the high dispersion of the HPA species makes it complicated to determine accurately the exact structure and actual number of water molecules within the HSiW crystalline structure. When the temperature increases above room temperature, between 25 and 150 °C, the diffractograms display the expected diffraction lines (*ca.* 10.5°, 15°, 18.5°, 21.3°, etc) for hexahydrated HSiW (H_4_SiW_12_O_40_·6H_2_O)^16^, which has a crystal structure similar to H_3_PW_12_O_40_·6H_2_O^16,33^. The diffractograms collected at temperatures between *ca.* 200 and 450 °C show a displacement of the reflections into a dehydrated phase, namely H_4_SiW_12_O_40_^16,34^. It is difficult to determine the actual phase of HSiW in HSiW/Al_2_O_3_ from the XRD data, probably due to the high dispersion of the HPA on the support^32,35,36^. Eventually, with the increasing temperature, the structure of the HSiW collapses leading to the formation of WO_3_, which is the only W-containing crystalline species (together with the supports) observed in the diffractograms recorded at 550 °C.

Concerning the supports, HSiW/X (X = TiO_2_, ZrO_2_, BN and CeO_2_) present the diffraction reflections of crystalline TiO_2_, ZrO_2_, BN, and CeO_2_ phases, respectively, in all the temperatures measured. However, HSiW/X (X = SiO_2_ and Al_2_O_3_) do not show the reflections of the supports (note that, in the case of silica, broad SiO_2_ reflections appear above 350 °C). The absence of diffraction lines for silica and alumina-supported HSiW catalysts has been reported by other authors, and it is attributed to a loss of crystallinity of the support after the impregnation process^32,35,36^.

The XRD analysis reveal that the crystalline structure of HSiW is partially preserved for all of the measured temperatures, until 550 °C. However, the high dispersion degree of HSiW species on the supports does not allow for an accurate identification of structure of the HSiW, more precisely the number of H_2_O molecules of crystallization, as well as the structure evolution with temperature.

As shown in Table 1, the crystallite size of the H_4_SiW_12_O_40_·6H_2_O phase at 150 °C increased from *ca.* 10 nm for HSiW/Al_2_O_3_ to *ca.* 24 and 29 nm for HSiW/TiO_2_ and HSiW/SiO_2_, respectively. The latter catalysts showing the highest values in the series. The surface areas (As) of HSiW/X calculated from Eq. 2 indicate the dispersion of HSiW in the catalysts. As observed in Table 1, the dispersion decreased in the order HSiW/SiO_2_ < HSiW/TiO_2_ < HSiW/ZrO_2_ < HSiW/BN < HSiW/CeO_2_ < HSiW/Al_2_O_3_.

**Figure 2:**
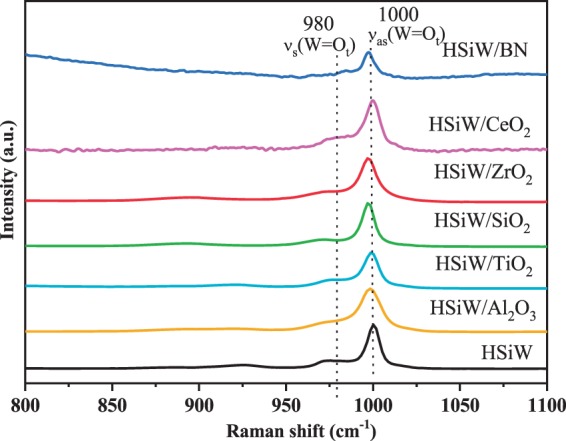
Raman spectra for the synthesized catalysts and the bulk HSiW.

Fig. 2 shows the Raman spectra recorded for the supported heteropolyacids and for bulk HSiW. All spectra show Raman bands at 1000 and 975 cm^−1^, assigned to ν_s_(W = O_t_) and ν_as_(W = O_t_), respectively^37^, which confirms that the heteropolyacid maintain the Keggin structure after the process of impregnation. The results obtained by Raman spectroscopy are in good agreement with the XRD analyses, even for the HSiW/Al_2_O_3_, and indicate that the HSiW structure is stable after deposition on the support.

**Figure 3:**
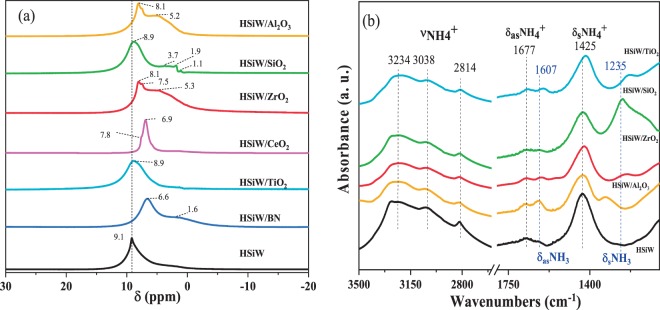
(**a**) ^1^H-NMR spectra of the HSiW based catalysts. (**b**) DRIFT spectra of NH_3_-adsorbed on supported and bulk HSiW.

The acidity of the catalysts was evaluated by ^1^H-NMR and DRIFT of NH_3_-adsorbed. ^1^H-NMR spectra of the catalysts are shown in Fig. 3. The peak at 9.1 ppm in the spectrum of bulk HSiW characterizes protons in anhydrous H_4_SiW_12_O_40_^37^. This peak can be observed in the spectra of all supported catalysts. However, both the actual position and the shape of the peaks are characteristic of the nature of the support, and therefore it can be used as a descriptor of the HPA-support interaction. Thus, the ^1^H-NMR peaks observed in the spectra for HSiW are almost identical to those of HSiW/SiO_2_ and HSiW/TiO_2_, indicating the lack of interactions between the protons of the HSiW and the TiO_2_ or SiO_2_ supports. On the other hand, the peaks in the spectra of HSiW/ZrO_2_, HSiW/BN, HSiW/CeO_2_, and HSiW/Al_2_O_3_ appear at lower chemical shifts than the peak of HSiW, suggesting the development of an interaction between the HSiW and the supports. The shifting of NMR peaks of supported HSiW *vs*. bulk HSiW has been observed previously and ascribed to the the weakening of the acidity of the protons in HSiW once they are supported^38^. As also observed in Fig. 3a, the spectra of HSiW/ZrO_2_ and HSiW/CeO_2_ display two peaks at 8.1 and 7.5 ppm which reveal the presence of two types of protons with different acidity. Peaks at lower shifts, ascribed to the supports, are also observed in the spectra of HSiW/SiO_2_ (at 1.1, 1.9, and 3.7 ppm)^37,39^, HSiW/ZrO_2_ (at 5.3 ppm)^40^, HSiW/Al_2_O_3_ (a broad signal around 5.2 ppm)^41^ and HSiW/BN (at 1.6 ppm)^42^. ^1^H-NMR results show that the acidity of protons in HSiW/TiO_2_ and HSiW/SiO_2_ is the same as in the bulk HSiW.

The nature of the aid sites in bulk and supported HSiW has been further investigated by DRIFT spectroscopy by using NH_3_ as a probe molecule. The 3500–1000 cm^−1^ region of the spectra of NH_3_ adsorbed on bulk and supported HSiW catalysts at 100 °C is shown in Fig. 3b. All catalysts show two set of bands ascribed to protonated ammonia on Brønsted acid sites namely ν(NH_4_^+^) bands at 3232, 3041 and 2809 cm^−1^, and δ_as_(NH_4_^+^) and δ_s_(NH_4_^+^) bands at *ca.* 1677 and 1425 cm^−1^, respectively^43–46^. The spectra of supported HSiW also show very weak bands due to ammonia adsorbed on Lewis acid sites at 1677–1620 and 1235–1331 cm^−1^ assigned to δ_as_(NH_3_) and δ_s_(NH_3_), respectively^43–45^. The identification of Lewis acid sites on HSiW/SiO_2_ is not possible because the bands overlap with the strong features of SiO_2_ between 1100 and 1350 cm^−1^. The spectrum for bulk HSiW shows no bands that can be attributed to Lewis acidity. It has been reported that the position of the δ_s_(NH_3_) band is sensitive to the Lewis acid strength of the adsorbing sites, shifting at higher frequencies for stronger Lewis acid sites^44^. The bands assigned to δ(NH_3_) appear at higher wavenumbers values in the spectra of HSiW/Al_2_O_3_ than in the spectra of HSiW/TiO_2_ and HSiW/ZrO_2_ suggesting a stronger acidity of the Lewis acid sites on HSiW/Al_2_O_3_.

### Catalytic performance for DME production

**Figure 4:**
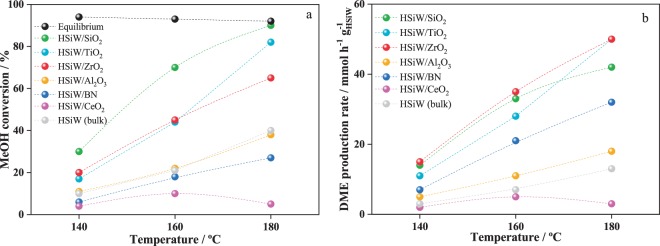
Evolution of methanol conversion (**a**) and DME productivity per gram of HSiW (**b**) with temperature for HSiW/X and bulk HSiW at 1 bar and 1.1 h^−1^. Catalysts pretreated at 220 °C in N_2_ for 1 h.

Bulk (HSiW) and supported (HSiW/X) samples were tested in the methanol dehydration reaction at 140, 160, and 180 °C and 1 bar; conversion values are reported after at least 4 hours on stream. Fig. 4 shows that all of the catalysts studied in this work have high activity for methanol dehydration at low temperatures; in fact, HSiW/SiO_2_ and HSiW/TiO_2_ reach methanol conversions close to the equilibrium at temperature as low as 180 °C. Importantly, the selectivity for all HSiW catalysts towards DME is 100%, irrespectively of reaction temperature. Although all HSiW catalysts display high activity, the actual conversion of methanol is characteristic of each catalyst, ranging between 90 and 10%. HSiW/SiO_2_, HSiW/TiO_2_, and HSiW/ZrO_2_ display the highest methanol conversions in the series, of *ca.* 90%, 80% and 60%, respectively. These values are higher than that of the bulk HSiW (*ca.* 40%). HSiW/Al_2_O_3_ shows similar methanol conversion than HSiW at all temperatures studied. Finally, BN and CeO_2_ supported HSiW show the lowest conversions in the series of *ca.* 25% and 5%, respectively. Fig. 4b shows the DME production rates normalized by the actual amount of HSiW on the catalysts. As observed, the catalysts showing the highest conversions, *i*.*e*., the ones supported on ZrO_2_, TiO_2_ and SiO_2_ also record the highest DME production rates, with HSiW/TiO_2_ and HSiW/ZrO_2_ showing the highest DME production rate of 50 mmol_DME_ h^−1^ g_HSiW_^−1^ at 180 °C.

These activity values are significantly higher than the values of state-of-the-art γ-Al_2_O_3_ and zeolites (HZSM-5 and HY), which display high activity at temperatures above 260 °C, being inactive below 200 °C^5,6,8,10,47^. Mesoporous aluminosilicates and aluminium phosphates have been also reported active methanol dehydration catalysts, but only reach methanol conversions higher than 50% at temperatures above 300 °C or 250 °C, respectively^10,47^. Other Lewis acid catalysts such as WO_3_/TiO_2_ or Nb_2_O_5_/TiO_2_ are known to have limited methanol conversion activity, below 10%, with DME selectivity ranging between 80–95%^12,13^. Only few articles describing the role of supported HPA as catalyst for methanol dehydration have been reported^15,16,23,26,48^. Alharbi *et al*. reported a methanol conversion of 13% over HSiW/SiO_2_ at 120 °C^48^, in line with 30% methanol conversion at 140 °C reported in our work. Ciftci *et al*.^15^ studied a H_3_PW_12_O_4_-based catalyst for the DME synthesis and found that methanol conversion increased with temperature, but decreases above 200 °C. Similar results were observed by Schnee *et al*., who reported that methanol conversion over bulk H_3_PW_12_O_4_ at 200 °C declines with TOS^49^. These results are in line with the trends observed in our work (see below).

A straightforward relationship between the catalytic activity and the BET specific surface areas of HSiW/X is not observed; the catalysts with the highest BET areas (Table 1), namely HSiW/CeO_2_ and HSiW/Al_2_O_3_ show lower activity than bulk HSiW, with HSiW/CeO_2_ showing the lowest activity in the series. On the other hand, SiO_2_, TiO_2_ and ZrO_2_, which presented the highest catalytic activities, have smaller BET specific surface areas (except HSiW/BN) than HSiW/CeO_2_ and HSiW/Al_2_O_3_. This observation supports the idea that surface catalysis is not the only main route for the dehydration of methanol to DME on supported HSiW. Noticeably, the activity trend displays an inverse correlation with the As values, and the catalysts showing lower dispersions, *i.e*., SiO_2_ and TiO_2_ supported HSiW, show the highest methanol conversions in the series. This feature indicates that the presence of HSiW aggregates is beneficial for the catalytic process, which is in good agreement with the predominant role of the pseudo-liquid catalysis. Note also that SiO_2_ and TiO_2_ supported HSiW catalysts are the ones showing the more acid protons (see ^1^H-NMR results), probably because the lower degree of dispersion of HSiW onto SiO_2_ and TiO_2_ results in a lower interaction between HSiW and the support, favoring proton mobility^50^. By contrary, by being well dispersed on the supports, a stronger interaction between HSiW and Al_2_O_3_, BN and Ce_2_O_3_ is developed, decreasing proton mobility and consequently the acidity of the catalysts, thus explaining the shifting of the ^1^H-NMR peaks to lower values observed in Fig. 3, resulting in less performance catalysts.

The acidity of the catalysts that displayed the highest activity, that is, those with methanol conversions higher than that of bulk HSiW, was quantified from NH_3_ adsorption isotherms. The densities of acid sites normalized to the mass of the catalyst and to mass of HSiW are summarized in Table 2. Note that the values obtained from the NH_3_ adsorption isotherms do not discriminate between the acid sites of the support and the HSiW.

**Table 2:** Density of acid sites of the supported HSiW catalysts.

Catalyst	Chemisorbed NH_3_ (mmol_NH3_ g_cat_^−1^)	Chemisorbed NH_3_ (mmol_NH3_ g_HSiW_^−1^)
Bulk HSiW	0.512	0.512
HSiW/TiO_2_	0.274	0.517
HSiW/SiO_2_	0.382	0.503
HSiW/ZrO_2_	0.209	0.454
HSiW/Al_2_O_3_	0.476	0.643

As shown in Table 2, the total acidity of the catalysts is characteristic of each sample, ranging between 0.209 mmol_NH3_/g_cat_ for HSiW/ZrO_2_ to 0.476 mmol_NH3_/g_cat_ for HSiW/Al_2_O_3_. These values are actually lower than that of bulk HSiW of 0.513 mmol_NH3_/g_cat_. The different number of acid sites of each catalyst appears to account for the different HSiW loading on each catalyst (see Table 1). Indeed, when normalized to the total HSiW content of each catalyst, all samples display a similar acidity of around 0.5 mmol_NH3_/g_HSiW_, which is close to that of bulk HSiW. HSiW/Al_2_O_3_ displays the highest density of acid sites in the series, probably due to the combined contribution of the acid sites of HSiW and of the support itself.

As shown in Fig. 4, the methanol dehydration activity (of the most active catalysts) follows the order HSiW/SiO_2_ > HSiW/TiO_2_ > HSiW/ZrO_2_, which is in line with the acidity (amount of NH_3_ chemisorbed per gram of catalyst) of the catalysts, except for bulk HSiW and HSiW/Al_2_O_3_. The latter catalysts show high acidity but low activity. The lower activity of the alumina-supported HSiW in comparison to TiO_2_ or SiO_2_-supported HSiW has already been reported before^51^. However, correlating the DME production activity of HSiW/Al_2_O_3_ with its acidity should be taken cautiously since, as explained above, the NH_3_ adsorption technique cannot be used to discriminate between acid sites associated to HSiW or Al_2_O_3_, but the latter acid site are not active for DME production from methanol at the low temperatures studied in this work. These findings indicate that not only the number of acid sites, but also the nature of the support and its interactions with the HPA, contribute to the observed catalytic performance. In fact, considering again the ^1^H-NMR spectra it seems like the catalysts that showed the weakest HSiW-support interactions are the ones that record the highest methanol conversions (HSiW/TiO_2_ and HSiW/SiO_2_).

**Figure 5:**
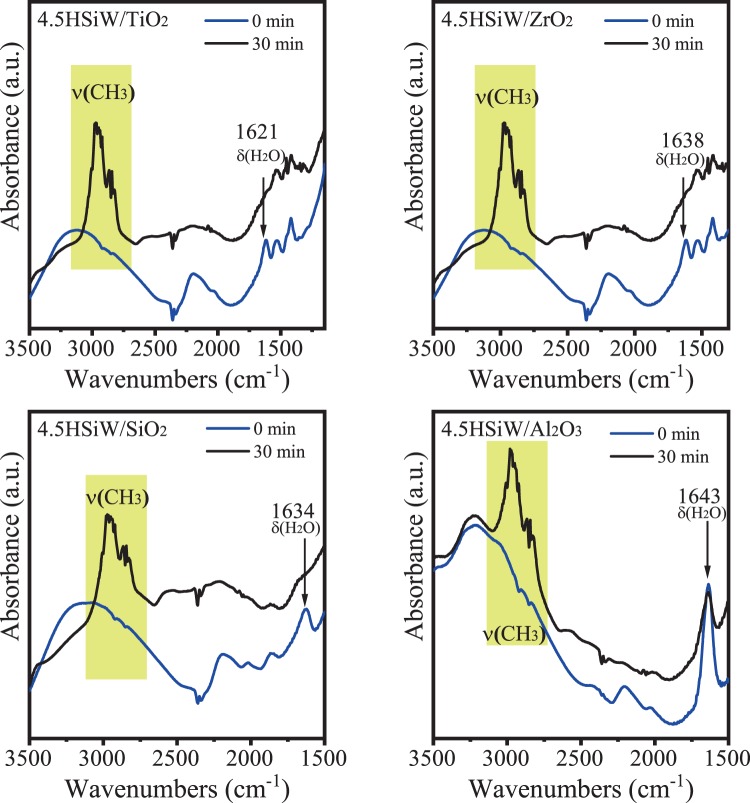
DRIFT spectra of the supported HSiW catalysts before and after 30 minutes of methanol flowing.

In addition, the accessibility of the methanol molecules to the heteropolyacid acid sites has been suggested to be critical for the catalytic performance. This feature can explain the highest methanol conversion activity observed with HSiW/SiO_2_, HSiW/TiO_2_, and HSiW/ZrO_2_ as compare to bulk HSiW despite the higher amount of acid sites in the latter. Complementary studies regarding the methanol adsorption mechanism were carried out by *in situ* DRIFTS with the aim to explain the behavior of the different supported catalysts. Fig. 5 shows the DRIFT spectra recorded before and after methanol adsorption on the catalysts.

After thermal treatment, and prior to methanol adsorption, all samples showed a band ascribed to the δ(H_2_O) mode of physisorbed water between 1616 and 1634 cm^−1^ ^52^. As observed in Fig. 5, this band disappeared from the spectra upon methanol admission into the cell, suggesting that methanol becomes adsorbed in the sites where H_2_O molecules were previously adsorbed. This pattern is observed in the spectra for HSiW/SiO_2_, HSiW/TiO_2_, and HSiW/ZrO_2_ but not in the case of HSiW/Al_2_O_3_. Indeed, the band ascribed to physisorbed H_2_O remains visible for the Al_2_O_3_ supported HSiW sample even after 30 minutes of methanol flow, suggesting a very strong retention of the H_2_O molecules that would prevent methanol from adsorbing and, consequently, reacting onto the HSiW/Al_2_O_3_. This explains the low conversion obtained with this catalyst, and suggests that the same situation might be happening with the HSiW/BN and HSiW/CeO_2_ catalysts.

### Effect of the reaction pressure for the synthesis of DME

As discussed above, HSiW/X catalysts are significantly more active (and selective) for the production of DME from methanol than the benchmarking catalysts γ-Al_2_O_3_ at atmospheric pressure and temperatures up to 180 °C. However, these reaction conditions are not suitable for the production of methanol from syngas, which take place at higher temperatures (T ≥ 220 °C) and pressures (P ≥ 10 bar). This feature is of an utter relevance for the direct synthesis of DME from syngas whereby syngas transformation to methanol and subsequently to DME should be carried out in a single reactor with bifunctional catalysts.

**Figure 6:**
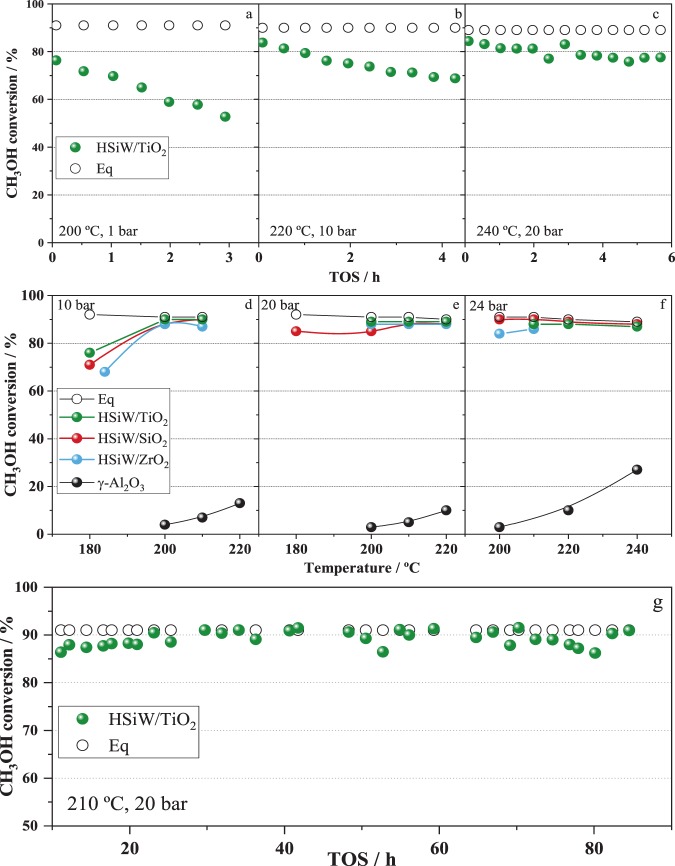
Methanol conversion obtained at different pressures and temperatures with the TiO_2_, SiO_2_ and ZrO_2_ supported HSiW catalysts. Equilibrium conversion and methanol conversion on γ-Al_2_O_3_ are shown. Pre-treatment: 1 h under N_2_ current at 220 °C.

In order to assess the effects of pressure and temperature for DME production with HSiW/X, we have studied the methanol dehydration reaction at high temperatures (up to 240 °C) and pressures (up to 24 bar) with SiO_2_, ZrO_2_ and TiO_2_ supported HSiW catalysts, since they showed the highest methanol conversions in the series. Catalysts supported on BN, CeO_2_ and Al_2_O_3_ were excluded from these experiments since they recorded lower methanol conversions than non-supported HSiW. As observed in Fig. 6a, raising the reaction temperature to 200 °C results in a severe decrease of methanol conversion over the HSiW/TiO_2_ catalysts from 76% to 53% after less than 3 hours on stream. The decrease in methanol conversion over HPA catalysts at 200 °C and 1 bar at increasing TOS has been also reported by other authors^15,49^. However, as observed in Fig. 6b, methanol conversion raises again to its initial value of around 78% by raising reaction pressure to 10 bar, while maintaining the temperature at 200 °C. A similar behaviour is observed for the ZrO_2_ and SiO_2_ supported HSiW catalysts, *i.e*., methanol conversion decreases at during time on stream at 200 °C and 1 bar, increasing back to its initial value by increasing reaction pressure to 10 bars. Increasing further reaction temperature to 220 °C (at 10 bar) results in a progressive (yet less severe than that observed at 200 °C, 1 bar) decline of the methanol conversion (Fig. 6c). Again, increasing reaction pressure to 20 bar allows increasing methanol conversion to its initial value of *ca.* 85% (Fig. 6d). Finally, a further increasing of reaction temperature to 240 °C (at 20 bar) leads to lower methanol conversions (Fig. 6e), that can be recovered by increasing the reaction pressure to 24 bar (Fig. 6f). This trend is observed for the three catalysts under study, HSiW/TiO_2_, HSiW/SiO_2_ and HSiW/ZrO_2_. Results of conversion at each condition in Fig. 6(b,d,f) were monitored for 5 hours and no decline in the activity was observed during that time. A similar trend in methanol conversion over HPA-based catalysts has been reported elsewhere: increasing conversions were obtained at temperatures from 150 to 200 °C, but further increase in the temperature resulted in a sharp decline in methanol conversion^15^.

The observed declining of activity with the increasing temperature (200 °C and above) can be ascribed to catalyst deactivation. The most typical cause of deactivation of acid catalysts during methanol (or in general alcohol) dehydration is the formation of coke deposits. Deactivation by coke formation is a progressive process that takes place at higher temperatures than those used in this work. For instance, it has been reported that no coke is formed over γ-Al_2_O_3_ at 230 °C upon DME adsorption^53^. Another extensive work on coking of zeolites during methanol conversion was carried out by Schulz, in which the temperatures considered relevant to study this process start at 270 °C^54^. These temperatures are higher than the ones explored in our work and we observed that methanol conversion decreases swiftly at temperatures as low as 200 °C (Fig. 6a). More importantly, catalyst deactivation by coke deposits is an irreversible process (unless coke is removed by thermal treatment under controlled atmosphere). As shown in Fig. 6d, methanol conversion at 200 °C increased almost immediately to its initial value, by increasing reaction pressure to 10 bar without affecting selectivity. Further increasing of reaction temperature to 220 °C and 240 °C resulted in a moderate declining of methanol conversions (Fig. 6b,c, respectively), which is recovered by increasing reaction pressure to 20 and 24 bar, respectively (Fig. 6e,f). Moreover, as observed in Fig. 6g, methanol conversion to DME (100% selectivity) remains stable for almost 100 h on stream, without any evidences of catalyst deactivation. These features show unambiguously that the loss of activity cannot be ascribed to catalyst deactivation by coke deposits. Taking into account that the high activity of HPAs (either supported or not) for methanol dehydration is due to the contribution of pseudo-liquid catalysis, it is reasonable to assume that the observed declining of the catalytic performance with T, should reflect the lack of contribution of the pseudo-liquid catalysis at high temperature and low pressure. In the pseudo-liquid catalysis, the system behaves as a gas-liquid system^19,20,55,56^, whereby methanol absorbs within the interpolyanion space between Keggin units. This process is favoured at low temperatures^19,57^, as generally the solubility of gases in liquids is lower as the temperature rises. The XRD data shown in Fig. 1 clearly reveal that increasing reaction temperature at 200 °C or higher, leads to the loss of crystallization water from HSiW, resulting in the formation of fully dehydrated phases. This feature could prevent the formation of the secondary structure of the HSiW (heteropolyanions + crystallization water), required for the pseudo-liquid catalysis to operate therefore resulting in the declining of catalytic activity. On the other hand, in agreement with Henry’s law, gas-liquid solubility also depends directly on the pressure of the system. The partial pressure of methanol is higher with the increasing pressure, and so it would be its solubility within the HSiW. The dependence of the absorption rate with the reaction pressure of molecules such as ethanol has been previously reported for this kind of solids^20,57^. The vision of this system as a gas-liquid one is consistent with the evolution of the methanol conversion observed in our results.

The results presented in this work demonstrate that HSiW based catalysts can be used for the production of DME from methanol at high temperatures (above 200 °C) provided the reaction is conducted at higher pressures. The pressure should be determined as a function of the temperature to allow the pseudo-liquid catalysis to take place.

## Conclusions

A series of supported HSiW catalysts were synthesized through the incipient wetness impregnation method. XRD and Raman analyses reveal that the Keggin structure of the HSiW remained unaltered after deposition on the supports. All catalysts show high activity for the dehydration of methanol to DME at low temperatures, especially those supported on ZrO_2_, TiO_2_, and SiO_2_, which are more active than bulk HSiW. This enhancement is ascribed to the better accessibility of the methanol to the active sites of the catalyst, enabling the interchange of crystallization water by the reacting molecule. The nature of the support is crucial in the performance of the supported HSiW; the development of strong support-HSiW interaction results in a lower mobility of the water located between the polyanions, preventing the contribution of the pseudo-liquid catalysis. Similarly, operating at temperatures above 180 °C prevent the access of methanol to the active sites between Keggin units and the loss of the pseudo-liquid character of the catalytic process. This effect can be avoided by increasing reaction pressure.

## Supplementary information

Supplementary information.
