# Supplementary material for: Effects of support and reaction pressure for the synthesis of dimethyl ether over heteropolyacid catalysts
Source: Sci Rep. 2020 May 22;10:8551. doi: 10.1038/s41598-020-65296-3 (PMC7244519; doi:10.1038/s41598-020-65296-3)
Supplement: Supplementary file 1 — Supplementary information. [file 41598_2020_65296_MOESM1_ESM.pdf]

Supplementary Information

Effects of support and reaction pressure for the synthesis of dimethyl ether over heteropolyacid catalysts

Cristina Peinado[1], Dalia Liuzzi[1], Rosa María Ladera-Gallardo[1], María Retuerto[1], Manuel Ojeda[1], Miguel A. Peña[1], Sergio Rojas[1]*

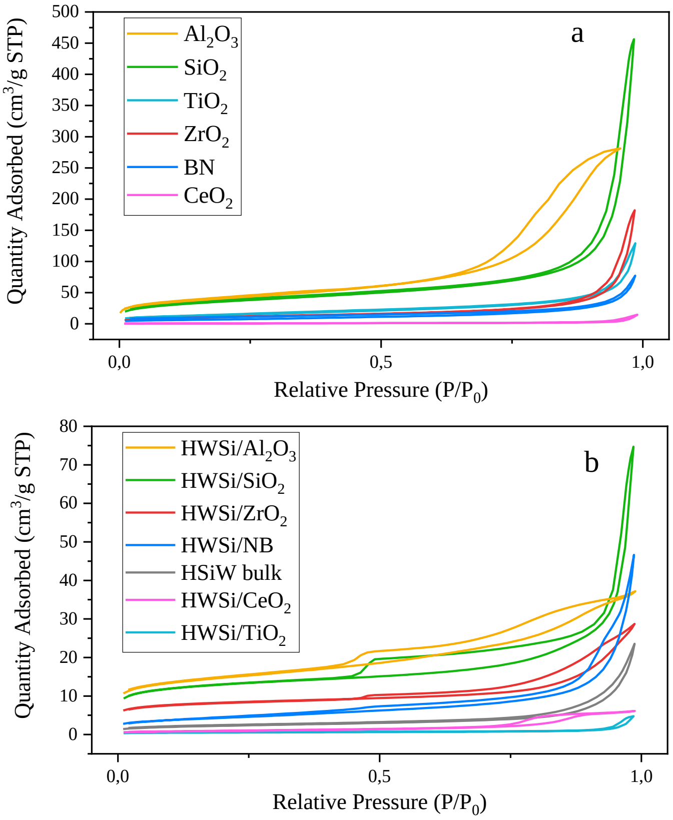

Supplementary Figure S1. N_2 absorption-desorption isotherms of supports (a) and catalysts (b)

The isotherms for the supports and catalysts are shown in Supplementary Figure S1 (a) and (b), respectively. The supports are non-porous materials with macropores showing type II isotherms with H3 hysteresis loops. Al_2O_3 shows as Type IV isotherm with H1 hysteresis.

The isotherms for the catalysts, Supplementary Fig. S1 (b) reveal a lower amount of adsorbed N_2. The isotherm of HSiW/SiO_2 is type II with H3 hysteresis. HSiW/CeO_2 shows a type IV isotherm with H1 hysteresis and HSiW/Al_2O_3 shows a type IV isotherm with H1 and H3 hysteresis.
